# Editorial: Methods and applications in striated muscle physiology

**DOI:** 10.3389/fphys.2022.979237

**Published:** 2022-08-11

**Authors:** Stefano Biressi, Qiang Ma, Norio Fukuda, Joseph J. Bass, Peter T. Wright

**Affiliations:** ^1^ Department of Cellular, Computational and Integrative Biology (CIBIO), University of Trento, Trento, Italy; ^2^ Department of Biopharmaceutics, School of Laboratory Medicine and Biotechnology, Southern Medical University, Guangzhou, Guangdong, China; ^3^ Department of Cell Physiology, The Jikei University School of Medicine, Tokyo, Japan; ^4^ MRC-Versus Arthritis Centre for Musculoskeletal Ageing Research, NIHR Nottingham Biomedical Research Centre, Centre of Metabolism, Ageing, and Physiology, School of Medicine, University of Nottingham, Derby, United Kingdom; ^5^ School of Life and Health Sciences, University of Roehampton, London, United Kingdom

**Keywords:** tecniques, innovation, skeletal, cardiac, muscle

Striated muscle exists in two types, skeletal and cardiac. They share numerous operational and structural characteristics, including sarcomere organization, with repeating functional units that confer the typical striated appearance observed in microscopic images of this tissue. Striated muscle is a highly adaptive and critically important metabolic tissue with intrinsic roles in health and disease. Striated muscle contraction supports various crucial physiological processes, including respiration, locomotion, posture (skeletal muscle), and pumping blood throughout the vascular system (cardiac muscle). Striated muscles are affected by many acquired and genetic pathophysiological conditions, which represent significant health and economic burden for modern society. Cardiac disorders, including ischemia and arrhythmias, are among the leading cause of death worldwide. Loss of skeletal muscle mass and strength is an indicator of neuromuscular genetic pathologies, aging, cancer, and a variety of chronic or acute conditions that are systemic or primarily affecting different tissues.

This Research Topic aims to describe new technical approaches and critically discuss existing methods and protocols applicable to the study of striated muscle physiology. The goal is to provide investigators with novel reliable tools: 1) to understand the fundamental mechanisms controlling striated muscle biology, 2) to characterize and diagnose striated muscle degenerative conditions, and 3) to identify innovative therapeutic strategies to preserve skeletal and cardiac muscle homeostasis and function.

One of the major limitations of the study of striated muscle characteristics for both basic research and diagnostic purposes is obtaining and preparing tissue for subsequent *ex vivo* molecular analysis or cellular and histological characterization. This is particularly critical if the donors of the biopsies are in a particularly fragile category, such as old people or individuals presenting coagulation defects, such as patients with chronic liver diseases. The Bergström biopsy is the gold-standard technique for sampling skeletal muscle and is used worldwide. This approach relies on collecting 25–125 mg of tissue with a 3–5 mm needle. Quinlan et al. report the results obtained with cohorts of patients affected by end-stage liver disease and non-cirrhotic non-alcoholic fatty liver disease undergoing percutaneous *vastus lateralis* biopsies with a Bergström needle. They report 90% of successful biopsies in over 71 attempts and only one adverse event (hematoma). This study also identifies the presence of subcutaneous adipose tissue as a critical aspect hindering productive sampling, indicating that appropriate patient selection and good technique mitigate against potential risks. An alternative to Bergström biopsy is microbiopsy, obtained with a needle of smaller diameter (typically 2–2.3 mm) and therefore characterized by decreased invasiveness. The size of tissue biopsy is generally smaller, ranging from 14 to 22 mg. Hester et al. show that percutaneous microbiopsy can be successfully performed in old individuals, and sufficient material is obtained for histological characterization of fiber types. This observation supports the idea that microbiopsies may be a valuable muscle sampling alternative to the Bergström technique, particularly for categories of fragile patients.

The obtainment and preparation of tissues for *ex vivo* investigation are also challenging for cardiac muscle. Cultivation of ventricular slices is increasingly used in basic and translational studies investigating mechanisms of ventricular dysfunction that lead to heart failure. Palmer and Bell describe, in the rat model, methods for removing and preparing cardiac papillary muscles and provide instruction for preparing cardiac slices for functional analyses. Klumm et al. describe a methodology that allows for long-term (up to 3 weeks) cultivation and functional analysis of beating human atrial myocardium. The possibility of establishing multicellular models of atrial myocardium expands the possibility of study beyond ventricular pathophysiology to discern atrial biology and atrial dysfunction.

The power of *in vitro* investigation in cardiac biology is disclosed by the methodological study reported by Krstic et al. They performed a visual characterization of beat-to-beat mitochondrial calcium fluxes in rat cardiomyocytes. For this purpose, they modify the fluorescent membrane-permeable Ca^2+^ indicator Rhod-2 by reducing it to di-hydroRhod-2, which is non-fluorescent unless it reaches the oxidative environment of mitochondria. This study highlights the need for optimized protocols to comprehend the complex mechanism underlying the contraction of striated muscle cells.

Calcium dynamics drive ATP production in the mitochondrial matrix, and play a critical role in the cytosol where Ca^2+^ governs striated muscle contraction and relaxation at the myofilament level via conformational modulations of the troponin complex. Rasmussen and Jin developed an approach using site-specific monoclonal antibodies as probes to monitor conformational changes of proteins. They applied this technique to study the Ca^2+^-binding subunit and the tropomyosin-binding/thin filament-anchoring subunits of troponin. Measurements at the sub-cellular level are instrumental in investigating striated skeletal and cardiac muscle contractility. In his manuscript, Marston reviews the current methods for measuring force production at the subcellular level, including single myofibril and single myofilament techniques. Herzog further critically discusses this area of expertise, particularly focusing on the isolation and testing of single sarcomeres. Also focused on sarcomeres is the publication by Adkins et al. that reports a quantification of the variability of *in vivo* sarcomere length measures obtained in human upper limb skeletal muscle with second harmonic generation microendoscopy. This state-of-the-art technique allows minimally invasive measures of sarcomere length. The assessment of the natural variability associated with this approach guides the development of robust experimental design and the interpretation of *in vivo* analyses of sarcomere length.

Skeletal muscle is not only composed of contractile syncytial muscle fibers but also other mononucleated cellular types, such as muscle stem cells, immune cells, interstitial and stromal progenitors, all reportedly playing a crucial role during muscle regeneration. Florio et al. disclose the potential use of electro-enhanced DNA transfer to murine skeletal muscle to investigate the characteristics of these mononucleated cells. Importantly, this approach can be applied not only to the study of healthy muscle but also to the investigation of alterations of the phenotypic properties that have been associated with defective repair and fibrosis in aging and dystrophic tissues.

The overarching theme of this Research Topic is to better understand striated tissue biology and uncover new diagnostic and therapeutic approaches to treat striated muscle disease. In this light, the discussion put forward by Sarvazyan in a review centered on valveless pumping based on the Liebau mechanism is particularly intriguing. Here, the author describes the biological occurrence of Liebau pumps, highlights the differences between Liebau pumping and the peristaltic flow, and discusses the potential uses and body sites that can benefit from implantable Liebau-type pumps.

In conclusion, this Research Topic on *Methods and applications in striated muscle physiology* incorporates novel original techniques and reviews of methodological approaches that can be used by investigators operating in the field of skeletal or cardiac muscle biology.

